# Estimates of Critical Upper Maxima (CT_max_ ) of Kelp Species Are Strongly Influenced by Biogeography and Thermal History

**DOI:** 10.1002/ece3.74276

**Published:** 2026-09-04

**Authors:** Tayla Leathers, Nathan G. King, Dan A. Smale

**Affiliations:** ^1^ The Laboratory Marine Biological Association of the United Kingdom Plymouth UK

**Keywords:** climate change, critical thermal maxima (CT_max_), kelp, macroalgae, ocean warming, physiology, plasticity, temperature, thermal limits, thermal performance curves

## Abstract

Experimentally derived critical thermal maxima values (CT_max_) are widely used to estimate species' thermal tolerances and, increasingly, to predict their vulnerability to chronic warming and heatwaves. However, accumulating evidence from model taxa such as fish and insects suggests that CT_max_ is dynamic and sensitive to multiple environmental and methodological factors, potentially limiting its application as a fixed species‐level metric. Here, we quantified CT_max_ in three habitat‐forming kelp species (*Laminaria digitata, Saccharina latissima* and *Laminaria ochroleuca* ) that occupy contrasting positions of their biogeographic range in the Western English Channel (northeast Atlantic). We assessed how methodological approach (whole plants vs. excised meristematic discs) and thermal history across multiple timescales (seasonal climatology and acute marine heatwaves, MHWs) shape upper thermal limits. Meristematic discs provided CT_max_ estimates comparable to whole plants in the two cool‐water species ( *L. digitata* and *S. latissima*), but significantly underestimated whole‐plant tolerance in the warm‐affinity species ( *L. ochroleuca* ). This divergence most likely reflected species‐specific biology with *L. ochroleuca* relying more strongly on whole‐organism integration and internal storage to maintain high thermal performance. Seasonal climatology influenced CT_max_ in both cool‐water species, with pronounced declines in autumn relative to spring and summer, consistent with narrower thermal safety margins and cumulative stress accrued over summer. Exposure to an extreme simulated MHW caused substantial reductions in CT_max_ across all species, with the largest decline (> 7°C) observed in *L. ochroleuca* . Together, these results show that CT_max_ is highly plastic, shaped by species' biological traits, methodological approach and thermal history. Treating CT_max_ as a fixed trait risks misrepresenting population‐level vulnerability of habitat‐forming kelp species and, most likely, other marine ectotherms. Incorporating thermal history and organismal biology into thermal‐tolerance assessments will improve predictions of the resilience of kelp species and the ecosystems they underpin, under future ocean warming.

## Graphical abstract

Experimentally derived critical thermal maxima (CTmax) are widely used to estimate species' thermal tolerance and increasingly to predict vulnerability to chronic warming. However, evidence shows how CTmax is dynamic and can be strongly influenced by multiple environmental and methodological factors, potentially limiting its application as a fixed species‐level metric to assess vulnerability. Here, we quantified CTmax in three habitat‐forming kelp species that occupy contrasting positions of their biogeographical range. We assessed how methodological approach and thermal history across multiple timescales (seasonal climatology and acute thermal stress) can shape upper thermal limits.

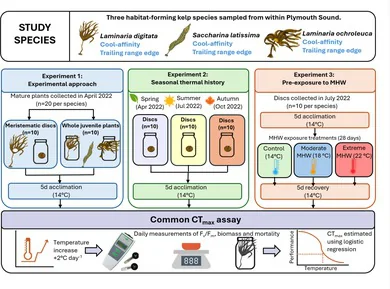

## 1 Introduction

Temperature exerts a strong influence over organismal biology and behaviour, constraining species biogeographic distributions to within a defined thermal niche in which they can function and persist (Martinez et al. 2015, Ørsted et al. 2022). Recent warming trends have shifted these thermal niches in both marine and terrestrial ecosystems, driving subsequent range shifts at a global scale (Chen et al. 2011; Poloczanska et al. 2013). Upper thermal limits are particularly important to define, as once temperatures exceed physiological limits, acute stress, reduced fitness, population declines and local‐to‐regional extinctions may follow (Smith et al. 2023; Vinagre et al. 2019). Whilst this may occur for all species, marine ectotherms in particular are highly sensitive to ocean warming trends, as they generally occupy a large proportion of their thermal niche, meaning that even small increases in environmental temperatures can exceed physiological tipping points (Sunday et al. 2012; Pinsky et al. 2019).

One approach to estimate species upper thermal limits is by experimental determination of their critical thermal maxima (CT_max_), that is, the highest temperature an organism can withstand during acute warming experiments before critical functions fail (Desforges et al. 2023). Measuring CT_max_ has a rich history in marine ecology and recent efforts have attempted to link estimates of CT_max_ with current and future thermal environments to predict vulnerability of species to ocean warming (Franco et al. 2018; Jordà et al. 2012; Sunday et al. 2012; Madeira et al. 2012). However, such approaches generally assume CT_max_ is a fixed trait, with a single critical upper temperature value applied across an entire species range, despite a range of factors that may influence experimentally‐derived estimates (Desforges et al. 2023). This simplification may limit our ability to accurately predict species vulnerability to warming (Smith et al. 2025).

The thermal environment that an organism experiences prior to experimental warming (i.e., thermal history) can strongly influence CT_max_ estimates. Where temperatures have been stressful, regulatory mechanisms may deplete energy reserves or cause acute cellular damage that reduces resilience for future challenges (Graiff et al. 2015; Straub et al. 2022). Conversely, less stressful exposures may induce physiological changes that harden or prime individuals (Bennett et al. 2022; Valladares et al. 2014; Hackerott et al. 2021; Nguyen et al. 2020). The strength and direction of these effects will largely depend on where a given population is located within the wider geographic distribution of the species (Harvey et al. 2022; Smith et al. 2024). Specifically, summer extreme temperatures are most likely to exceed thermal safety margins (i.e., the difference between a species' upper thermal limit and the maximum temperatures it regularly experiences) at a species' warm range edge and be less stressful or benign at cooler parts of its range (Wiens 2016; Sunday et al. 2014).

Importantly, the influence of thermal history is likely to be mediated by the timescale over which it occurs. Organisms experience temperature variability across multiple temporal scales, such as rapid increases associated with marine heatwaves (MHWs), unfolding over days to weeks (Hobday et al. 2016) and thermal stress related to seasonal climatology that accumulates over weeks to months (Chapple et al. 1998), with these differing timescales activating distinct physiological pathways (Kefford et al. 2022). Intraspecific variability in thermal tolerance has been demonstrated across a range of taxa, linked to factors such as life history stage and latitudinal distribution (Smith et al. 2025). Therefore, we need to understand not only how CT_max_ shifts in response to thermal history, but also how these shifts interact with the location of a population within the species' wider latitudinal distribution, as this interplay ultimately determines vulnerability to ocean warming.

Marine macrophytes, such as seaweeds and seagrasses, support critical ecosystem functioning, enhancing biodiversity and contributing to essential nutrient cycling (Teagle and Smale 2018; Pessarrodona et al. 2018). Kelp species, in particular, represent important coastal foundation organisms, being widely distributed across temperate and subpolar regions and underpinning highly productive and diverse habitats along rocky shores (Smale et al. 2013; Teagle and Smale 2018). As sessile organisms, their distribution and performance is heavily constrained by local temperatures (Jayathilake and Costello 2020; Eggert 2012), and recent range shifts and local extinctions have been linked to increases in mean and extreme temperatures across the global ocean (Wernberg et al. 2024; Smale 2020). These ecological and physiological sensitivities make kelps excellent model organisms for examining how CT_max_ varies across species, methods and thermal histories, and for understanding the ecological implications of shifts in upper thermal limits.

A specific challenge in kelp and broader seaweed thermal tolerance research is, however, the commonplace use of reduced tissue representations in acute warming experiments. Because mature kelp sporophytes (hereafter ‘plants’) are generally large and structurally complex, many studies experiment on excised meristematic discs rather than whole plants to increase replication, logistical feasibility and experimental control (Geange et al. 2021; Smillie et al. 1987). However, kelp species differ markedly in the extent to which meristematic tissues depend on whole‐plant integration for physiological buffering, nutrient redistribution and access to carbon reserves (Hurd et al. 2014). As a result, disc‐based assays may accurately represent whole‐plant thermal tolerance in some kelps, yet misrepresent it in others due to species‐specific differences in tissue integration, photochemical behaviour or resource storage (Sharma et al. 2014; Enríquez et al. 2002; Geange et al. 2021). Despite their widespread use in experimental seaweed ecophysiology, direct comparisons between discs and whole plants remain rare, meaning that the reliability of this common approach must be evaluated on a species‐by‐species basis.

In this study, we estimated CT_max_ in three habitat‐forming kelp species (with overlapping latitudinal distributions and contrasting thermal tolerances) to address the following key questions: (1) How representative is the commonly used methodological approach of using plant fragments (meristematic discs) rather than whole plants, for experimentally determining CT_max_? (2) How does thermal history across different timescales (i.e., seasonal conditions vs. acute marine heatwaves) shape CT_max_, and does the magnitude or direction of these effects vary depending on a species' position within its broader thermal range? The overall objective of the study was to compare CT_max_ estimates between species, methodological approaches and thermal history, to shed light on the variability in CT_max_ and how useful this metric is for predicting vulnerability to current and future ocean warming.

## 2 Methods

### 2.1 Study Species and Context

Experimental work was conducted on three habitat‐forming kelp species (*Laminaria digitata, Saccharina latissima* and *Laminaria ochroleuca* ), sampled from within Plymouth Sound (Firestone Bay; 50.361096, 4.159963) in 2022, which is located in the Western English Channel (WEC) region within the wider northeast Atlantic. The WEC represents a biogeographical transition zone for kelp, where a number of species are situated at different parts of their latitudinal range (Smale et al. 2013). *Laminaria digitata* and *Saccharina latissima* are cold‐temperate species, exhibiting optimum growth and survival between 10°C and 15°C (Bolton and Lüning 1982; Tom Dieck 1993). Both species are located towards the warm portion of their distribution in the study region, and have experienced range contractions in northern Europe in response to gradual ocean warming (Raybaud et al. 2013; Moy and Christie 2012). *Laminaria ochroleuca* is a relatively warm‐adapted species, which has a thermal optimal range between 12°C and 18°C (Biskup et al. 2014; Izquierdo et al. 2002; Franco et al. 2018) and has expanded its range within the study region (Hargrave et al. 2017; Smale et al. 2015; Teagle and Smale 2018). Temperatures in the WEC have increased considerably in recent decades (Kassem et al. 2022; Simon et al. 2023) and have been linked to shifts in the structure of kelp‐dominated habitats, a trend that is set to continue under ocean warming (Assis et al. 2018; Taylor‐Robinson et al. 2025; Salland et al. 2026). Based on known latitudinal distributions and thermal tolerances, we expected CT_max_ estimates would be higher for *Laminaria ochroleuca* compared to the two cooler affinity species, being less affected by summertime thermal stress and pre‐exposure to MHWs.

### 2.2 CT_max_ Assays

There is no standardised approach for measuring CT_max_, as it can be determined using different end points and differing rates of thermal ramping. Indeed, differences in experimental duration can significantly impact estimates of upper thermal limits (Ørsted et al. 2022; Bates and Morley 2020; Jørgensen et al. 2019). Selecting biologically relevant ramping rates that yield CT_max_ values that are representative of a species' functional thermal niche is critical. A ramping rate over the course of days (rather than minutes or hours) is more ecologically relevant as it is comparable to rates of onset of actual MHWs experienced by natural populations (Holbrook et al. 2020). In all experiments here, we used the same CT_max_ assay (Figure S1), whereby temperatures were increased by 2°C day^−1^ and response variables were measured daily. All CT_max_ trials were conducted in a 1000 L tank, held at 14°C prior to ramping, with light provided through LED lighting tiles (AquaRay) under a photosynthetic flux density of ~120 μmol m^−2^ s^−1^ and a 12:12 light–dark regime. Salinity and temperature were monitored daily, maintained between 33 and 35 ppt and ±0.5°C. Within the 1000 L tank, individuals were held in independent 2 L aquaria and were continuously aerated with air stones, and water changes were conducted every 3 days with locally sourced seawater.

### 2.3 Thermal Response Parameters

*F*_v_/*F*_m_ represents the maximum quantum yield of photosystem II (PSII) and is widely accepted as an efficient indicator of stress in macroalgae. After 15 min of dark acclimation, *F*_v_/*F*_m_ was measured using Pocket PEA chlorophyll fluorimeter (Hansatech Instruments). Values > 0.6 are generally considered indicative of healthy tissue, whereas values below this indicate increasing stress due to damage to the photosynthetic apparatus or photoinhibition. Change in biomass (%) was also measured as a stress indicator, whereby samples were weighed at each temperature timepoint to determine growth or tissue loss under increasing thermal stress. Mortality was determined where *F*_v_/*F*_m_ values of ≤ 0.1, indicating that the tissue is no longer photosynthetically viable and therefore used as a proxy for the individuals' ‘end‐point’.

### 2.4 Quantifying CT_max_

For each individual, a four‐parameter log‐logistic response model was fit using the drc package in R (Ritz et al. 2016) to derive CT_max_ from fitted parameters. The threshold for CT_max_ was defined as the temperature where the model predicted a decline of 80% performance, where *F*_v_/*F*_m_ fell below 0.2 (Baker 2008). Three models were used, testing main effects versus more complex interactions, and the most appropriate model was selected based on the lowest Akaike Information Criterion (AIC). All models and their outputs can be seen in Table S1. To determine the uncertainty of selected models, a total of 500 bootstrap iterations were carried out for each species and treatment, allowing differences between modelled and calculated parameters to be evaluated (Canty and Ripley 2025).

#### 2.4.1 Question 1: Experimental Approach

##### 2.4.1.1 Experiment 1: Discs Versus Whole Plants

Excising discs of tissue has been routinely used in experimental seaweed ecophysiology, as a surrogate to represent whole plant responses (Diehl et al. 2021; Leathers et al. 2023; Hereward et al. 2020; Niedzwiedz et al. 2022). However, direct comparisons have not been made, potentially compromising the ecological validity of studies adopting this approach. In April 2022, a single meristematic disc (diameter = 3.0 cm, area = 7.1 cm^2^) was excised from ten separate mature plants and compared with responses of ten whole, juvenile sub canopy plants in concurrent CT_max_ trials (see above). Individuals were maintained at ambient temperatures (i.e., local sea water temperature at time of collection) for 5 days to acclimatise to experimental conditions prior to CT_max_ trails.

#### 2.4.2 Question 2: Effects of Thermal History Across Different Timescales

##### 2.4.2.1 Experiment 2: Seasonal Thermal History

To examine seasonal variability in CT_max_, mature individuals were collected during spring (April), summer (July) and autumn (October) 2022. A single meristematic disc (as described above) was excised from ten separate mature plants of our three kelp species. Discs were placed in independent 2 L aquaria and submerged in a 100 L holding tank for a five‐day acclimatisation period before commencing the CT_max_ trails.

##### 2.4.2.2 Experiment 3: Acute Thermal History (Pre‐Exposure to Marine Heatwaves)

To assess the effect of acute thermal history on CT_max_, individuals were exposed to an environmentally relevant simulations MHW prior to CT_max_ trials. In July 2022, a single meristematic disc (as described above) was excised from ten separate mature plants of the three kelp species. Discs were placed into 2 L aquaria sat in 64 L water baths, used to manipulate the temperature of the system. Discs were maintained at ambient temperatures for 5 days to acclimatise to experimental conditions. Temperatures were then increased by 1°C day^−1^ until desired MHW temperatures were reached. The experimental intensities for the MHW followed the framework of Hobday et al. (2018) and consisted of: control (14°C: ambient, annual average seawater temperature), moderate (18°C: 1–2 × upper 90th percentile − climatological mean) and extreme (22°C: 4–5 × upper 90th percentile—climatological mean). The MHW period lasted for 28 days, reflecting a realistic but long lasting MHW, after which temperatures were lowered 1°C day^−1^ to ambient for a recovery period of 5 days. After the recovery period, CT_max_ trials (see above) were conducted on surviving discs.

### 2.5 Statistical Analysis

Statistical analysis was conducted in RStudio in which each species was analysed separately. Differences in the mean of response variables, *F*_v_/*F*_m_, change in biomass and mortality was tested for each experiment using a two‐way repeated measures analysis of variance (ANOVA), using the ‘anova’ function in the rstatix package. For each experiment, the model had two factors (i.e., Temperature and Approach, Temperature and Season and Temperature and MHW Exposure). Statistical differences were examined at each temperature point (14°C–32°C) between each level in the experiment (Experiment 1, 2 levels: Discs, Plant; Experiment 2, 3 levels; Spring, Summer, Autumn and Experiment 3, 3 levels; No MHW, Moderate MHW (18°C), Extreme (22°C)). Normality of data was tested using Shapiro‐Wilks test, in which a *p* value > 0.05 indicated normality. Where the assumption of sphericity was violated (Mauchlys test) degrees of freedom were corrected, using Greenhouse–Geisser estimates of Sphericity. Where significant interactions were detected (*p* < 0.05), *post hoc* pair‐wise comparison were conducted to identify where differences lay.

Differences in CT_max_ between were evaluated using a one‐way ANOVA. For the experimental approach trial, the factor of interest comprised two levels (discs vs. whole plants), for seasonal variability three levels (spring, summer and autumn) and for pre‐exposure to MHWs three levels (control, moderate and extreme). When significant effects were detected (*p* < 0.05), *post hoc* (Tukey's test) pair‐wise comparisons were conducted to identify where differences lay.

## 3 Results

### 3.1 Experimental Approach (Discs vs. Whole Plants)

**FIGURE 1:**
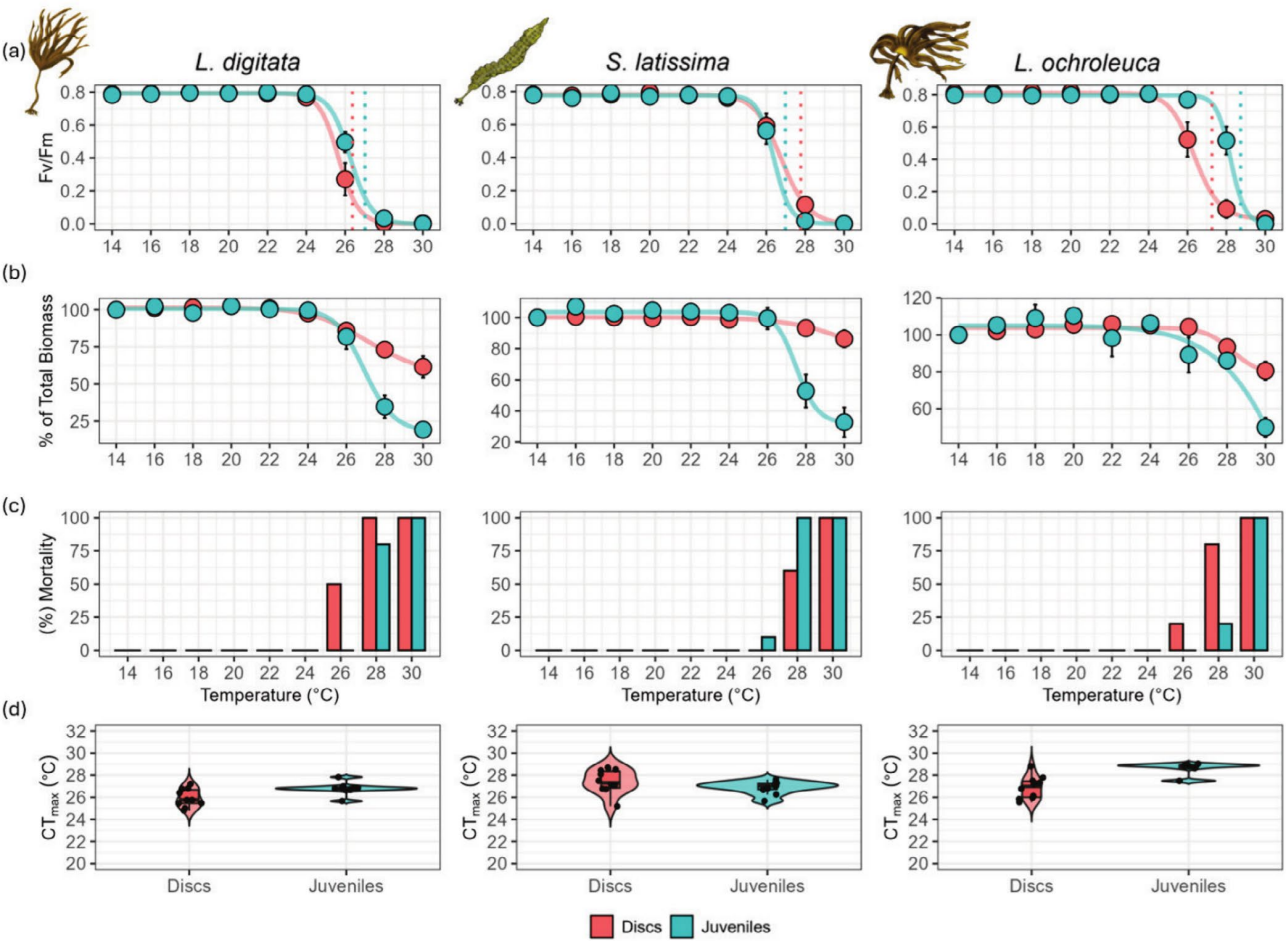
CT_max_ stress response assays for meristematic discs and whole juvenile plants of the cool‐affinity species *L. digitata* and *S. latissima* and the warmer affinity species, *L. ochroleuca* . Mean (±SE) (a) *F*_v_/*F*_m_ values with CT_max_ indicated with a dashed line, (b) change in biomass (%), (c) proportion of sample considered ‘dead’ (*F*_v_/*F*_m_ = ≤ 0.1) and (d) CT_max_ estimates.

For the cool‐water species *Laminaria digitata* , discs and whole plants showed very similar trajectories of thermal stress. *F*_v_/*F*_m_ remained high for both discs and whole plants at lower temperatures, between 14°C and 24°C and began to decline at approximately the same threshold (~26 C, Figure 1). Biomass loss and the onset of mortality also occurred at similar temperatures, with discs showing only a slightly earlier decline. A comparable pattern was observed in *Saccharina latissima*, where discs and whole plants exhibited nearly identical *F*_v_/*F*_m_ declines up to ~26°C–28°C (Figure 1). For both *L. digitata* and *S. latissima*, although whole plants tended to exhibit complete mortality slightly earlier than discs, discs adequately captured whole‐organism thermal physiological stress using *F*_v_/*F*_m_ patterns in these cool‐affinity species, in which discs and whole plants were not statistically different (Table 1).

**TABLE 1:** Results of repeated measures ANOVA to test for differences in *F*_v_/*F*_m_, change in biomass and mortality for the cool‐affinity species, *L. digitata* and *S. latissima*, and the warmer‐affinity species, *L. ochroleuca* for (a) Tissue (Experiment 1), (b) Seasonal thermal history (Experiment 2) and (c) Acute thermal history (Experiment 3).

(a) Discs versus whole plants	*L. digitata*			*S. latissima*			*L. ochroleuca*		
Effect	df	*F*	*p*	df	*F*	*p*	df	*F*	*p*
*F*_v_/*F*_m_									
Treatment	1	3.29	0.103	1	1.80	0.213	**1**	**9.82**	**0.012**
Temperature	8	426.71	**< 0.001**	8	244.24	**< 0.001**	8	155.71	**< 0.001**
Treatment: Temperature	8	2.72	0.130	8	0.81	0.429	8	9.75	**0.001**
Change in biomass (%)									
Treatment	1	13.99	**0.005**	1	13.41	**0.005**	1	1.99	0.191
Temperature	8	89.85	**< 0.001**	8	31.57	**< 0.001**	8	22.89	**< 0.001**
Treatment: Temperature	8	12.86	**< 0.001**	8	20.90	**< 0.001**	8	3.55	**0.014**
Mortality (%)									
Treatment	1	10.76	**0.01**	1	9.00	**0.01**	1	10.29	**0.011**
Temperature	8	134.23	**< 0.001**	8	159.75	**< 0.001**	8	70.55	**< 0.001**
Treatment: Temperature	8	5.82	**< 0.001**	8	4.24	**< 0.001**	8	8.76	**< 0.001**

(b) Seasonal thermal history	*L. digitata*			*S. latissima*			*L. ochroleuca*		
Effect	df	*F*	*p*	df	*F*	*p*	df	*F*	*p*
*F*_v_/*F*_m_									
Treatment	2	49.08	**< 0.001**	2	35.61	**< 0.001**	2	4.92	**0.02**
Temperature	8	264.75	**< 0.001**	8	177.69	**< 0.001**	8	168.35	**< 0.001**
Treatment: Temperature	16	12.38	**< 0.001**	16	16.18	**< 0.001**	16	2.52	**0.002**
Change in biomass (%)									
Treatment	2	46.97	**< 0.001**	2	30.16	**< 0.001**	2	15.52	**< 0.001**
Temperature	8	107.77	**< 0.001**	8	95.34	**< 0.001**	8	49.47	**< 0.001**
Treatment: Temperature	16	14.42	**< 0.001**	16	22.03	**< 0.001**	16	8.27	**< 0.001**
Mortality (%)									
Treatment	2	22.57	**< 0.001**	2	22.71	**< 0.001**	2	2.37	0.122
Temperature	8	167.79	**< 0.001**	8	74.27	**< 0.001**	8	174.72	**< 0.001**
Treatment: Temperature	16	7.22	**< 0.001**	16	7.26	**< 0.001**	16	1.20	0.274

(c) Acute thermal history	*L. digitata*			*S. latsissima*			*L. ochroleuca*		
Effect	df	*F*	*p*	df	*F*	*p*	df	*F*	*p*
*F*_v_/*F*_m_									
Treatment	2	11.76	**0.008**	2	9.39	**0.03**	2	22.76	**< 0.001**
Temperature	8	65.89	**< 0.001**	8	23.56	**< 0.001**	8	51.96	**< 0.001**
Treatment: Temperature	16	5.57	**< 0.001**	16	1.47	0.1	16	6.63	**< 0.001**
Change in biomass (%)									
Treatment	2	9.72	**0.01**	2	2.06	0.243	2	26.59	**< 0.001**
Temperature	8	42.32	**< 0.001**	8	28.98	**< 0.001**	8	32.66	**< 0.001**
Treatment: Temperature	16	5.85	**< 0.001**	16	0.59	0.861	16	8.49	**< 0.001**
Mortality (%)									
Treatment	2	7.37	**0.002**	2	4.00	0.11	2	18.67	**< 0.001**
Temperature	8	42.31	**< 0.001**	8	14.29	**< 0.011**	8	43.27	**< 0.001**
Treatment: Temperature	16	3.00	**< 0.001**	16	1.49	0.165	16	8.97	**< 0.001**

*Note:* Significant values (*p* < 0.05) are indicated in bold.

In contrast, clear differences across all responses emerged in the warm‐water species *Laminaria ochroleuca* (Figure 1, Table 1). Discs showed earlier declines in *F*_v_/*F*_m_ (beginning around 24°C–26°C) and reached mortality thresholds sooner than whole plants. Whole plants retained higher physiological performance and remained viable until higher temperatures (~28°C–30°C). Biomass loss was variable but broadly similar, whereas complete mortality occurred at 28°C in discs compared to 30°C in whole plants. Overall, this indicates that excised discs under‐represent the thermal tolerance of the warm‐adapted species.

#### 3.1.1 CT_max_ Estimates

The logistic regression model revealed that the magnitude of CT_max_ differences between tissue types depended strongly on species thermal affinity (Table 3). There was no significant difference between discs and whole plants (Table 2) in both *L. digitata* (26.3°C ± 0.3°C vs. 27.8°C ± 0.6°C) and *S. latissima* (27.7°C ± 0.8°C vs. 27.0°C ± 0.1°C). In contrast, CT_max_ differed significantly between discs and whole plants (Table 2) for *L. ochroleuca* (27.5°C ± 0.4°C vs. 28.7°C ± 0.3°C).

**TABLE 2:** Results of a one‐way ANOVA to test for significant differences in CT_max_ estimates between experimental treatments, seasonal sampling and pre‐exposure to MHWs, for the cool‐affinity species *L. digitata* and *S. latissima*, and the warmer affinity species, *L. ochroleuca* .

	*L. digitata*			*S. latissima*			*L. ochroleuca*		
	df	*F*	*p*	df	*F*	*p*	df	*F*	*p*
Discs versus whole plants	1	0.617	0.231	1	0.256	0.619	1	9.197	**0.007**
Residuals	18			18			18		
Seasonal thermal history	2	14.68	**< 0.001**	2	9.653	**< 0.001**	2	0.693	0.509
Residuals	27			27			27		
Acute thermal history	2	7.293	**0.007**	2	3.826	**0.036**	2	11.93	**< 0.001**
Residuals	13			24			15		

*Note:* Significant results (*p* < 0.05) are indicated in bold.

### 3.2 Effects of Thermal History Across Different Timescales

#### 3.2.1 Seasonal Thermal History

**FIGURE 2:**
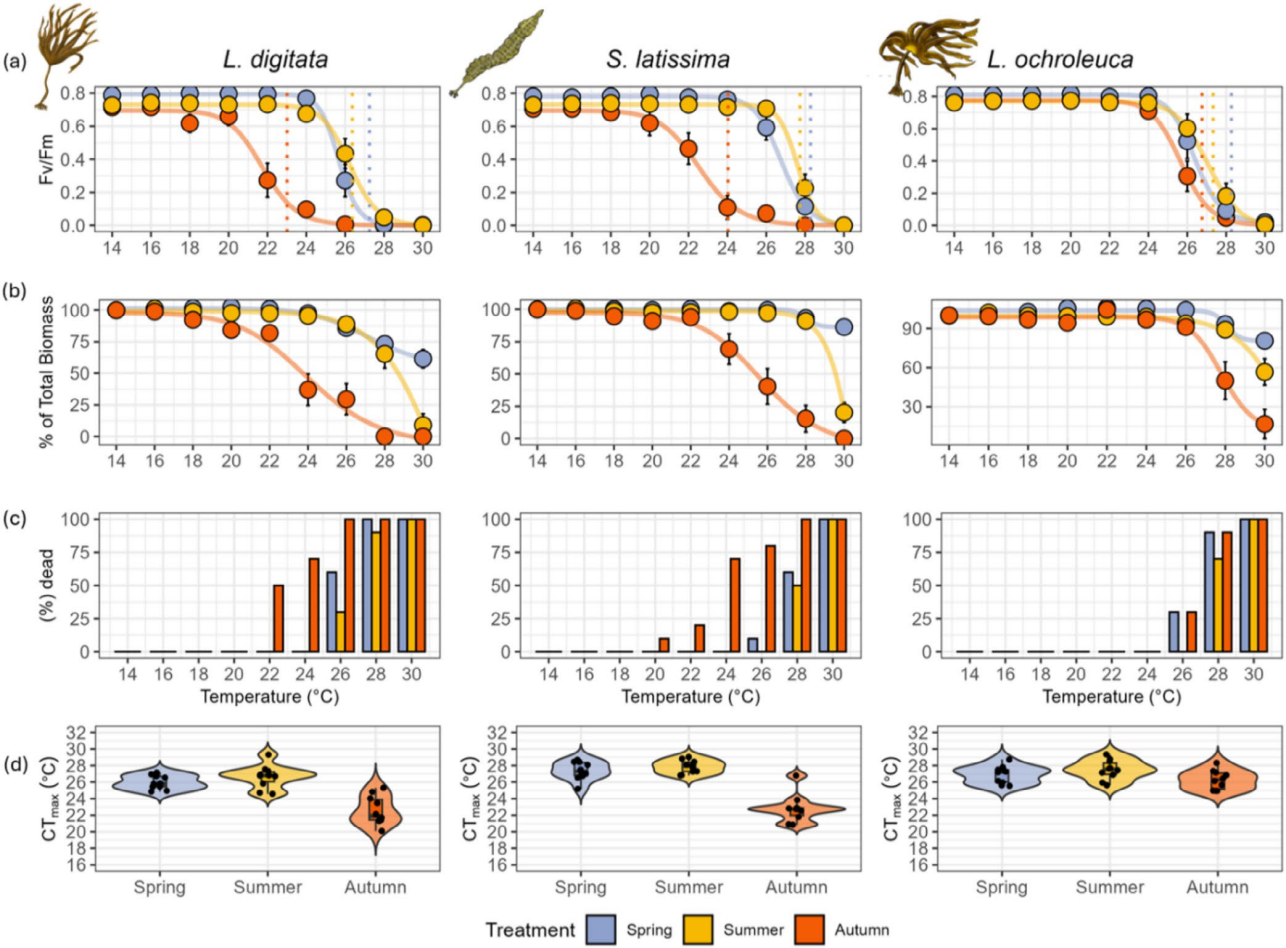
CT_max_ stress response assays for samples of cool affinity species *L. digitata* and *S. latissima*, and warm affinity species *L. ochroleuca* tested in spring, summer and autumn timepoints. Mean (±SE) (a) *F*_v_/*F*_m_ values with CT_max_ indicated with a dashed line, (b) change in biomass (%), (c) proportion of sample considered ‘dead’ (*F*_v_/*F*_m_ = ≤ 0.1) and (d) CT_max_ estimates.

Seasonality had a clear influence on thermal stress responses across all three kelp species (Table 1), with individuals collected in autumn consistently showing reduced performance compared to those collected in spring and summer (Figure 2). However, the strength of this effect depended on the thermal affinity of the species.

In *L. digitata* , *F*_v_/*F*_m_ remained around 0.7 for spring and summer samples until ~24°C–26°C, after which both cohorts exhibited sharp declines (Figure 2). In contrast, autumn samples began to show declines at considerably lower temperatures (~20°C–22°C), indicating earlier onset of thermal stress. Biomass loss followed a similar pattern with both spring and summer tissues maintaining structure until ~28°C–30°C, whereas autumn samples experienced measurable tissue loss at lower temperatures. Mortality occurred sooner in autumn with 50% of samples deemed non‐viable by 22°C. *S. latissima* showed a similar pattern in that spring and summer samples retained high *F*_v_/*F*_m_ values until ~24°C–26°C, followed by steep declines at higher temperatures (Figure 2). Autumn samples again showed earlier physiological deterioration, with pronounced declines in *F*_v_/*F*_m_ occurring at ~20°C. Similarly, biomass loss and mortality occurred at lower temperatures in autumn relative to spring and summer.

For the warm‐affinity species *L. ochroleuca*, seasonal differences were less pronounced. *F*_v_/*F*_m_ remained high in all seasons until ~26°C, after which autumn samples declined more steeply than those collected in spring and summer (Figure 2). Biomass loss and mortality occurred slightly earlier in autumn samples, but the magnitude of seasonal divergence was substantially smaller than observed in the cool‐water species.

##### 3.2.1.1 CT_max_ Estimates

The logistic regression model revealed substantial seasonal shifts in CT_max_ for the two cool‐affinity species (Table 3). In *L. digitata* , CT_max_ was significantly lower in autumn (22.9°C ± 0.4°C) compared to spring (26.3°C ± 0.3°C) and summer (27.3°C ± 0.3°C). A similar but more pronounced pattern occurred in *S. latissima*, where C_Tmax_ declined by > 4°C, and was significantly lower in autumn (24.0°C ± 0.8°C) compared to both spring (27.7°C ± 0.2°C) and summer (28.3°C ± 0.1°C). In contrast, the warm‐affinity species *L. ochroleuca* showed comparatively modest seasonal variation, with CT_max_ decreasing from 27.5°C ± 0.1°C in spring and 28.2°C ± 0.1°C in summer to 26.8°C ± 0.1°C in autumn, but differences were not significant (Table 2).

**TABLE 3:** Estimates CT_max_ values from the thermal performance curves of *F*_v_/*F*_m_ of the cool affinity species *L. digitata* and *S. latissima*, and the warm affinity species *L. ochroleuca* under different experimental treatments.

Treatments	*L. digitata*		*S. latissima*		*L. ochroleuca*	
	CT_max_°C	SE	CT_max_°C	SE	CT_max_°C	SE
Tissue						
Discs	26.3	0.0303	27.7	0.0890	27.5	0.0126
Juveniles	27.0	0.0687	27.0	0.0162	28.7	0.0121
Season						
Spring	26.3	0.0428	27.7	0.0245	27.5	0.0319
Summer	27.3	0.0212	28.3	0.0185	28.2	0.0417
Autumn	22.9	0.0443	24.0	0.0114	26.8	0.0373
MHW Exposure						
No MHW	27.1	0.548	28.2	0.0979	28.3	0.0315
Moderate MHW	27.8	0.0692	28.2	0.0367	27.8	0.0084
Extreme MHW	24.4	0.0548	26.5	0.0339	20.2	0.0499

#### 3.2.2 Acute Thermal History (Pre‐Exposure to MHWs)

**FIGURE 3:**
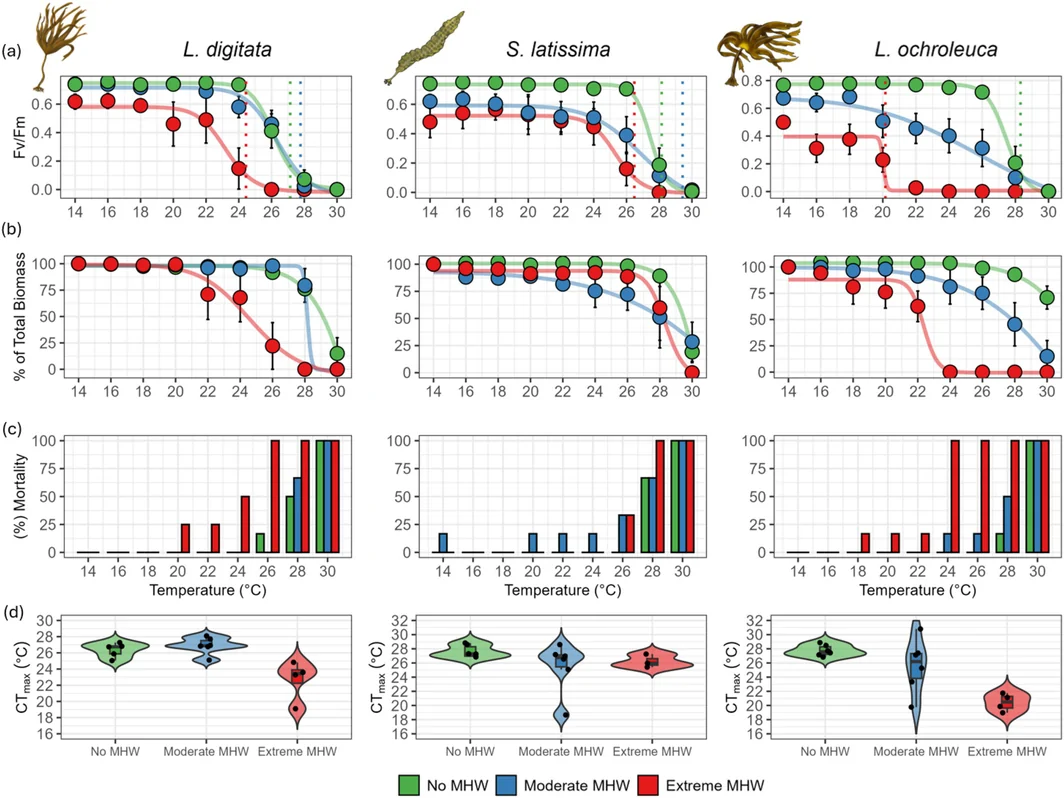
CT_max_ stress response assays for samples of the cool affinity species *L. digitata* and *S. latissima*, and the warm affinity species *L. ochroleuca* , after exposure to No MHW (14°C), Moderate MHW (18°C) and Extreme MHW (22°C) treatments. Mean (±SE) (a) *F*_v_/*F*_m_ values with CT_max_ indicated with a dashed line, (b) change in biomass (%), (c) proportion of sample considered ‘dead’ (*F*_v_/*F*_m_ = ≤ 0.1) and (d) CT_max_ estimates.

Across all three kelp species, prior exposure to simulated MHWs reduced thermal performance relative to control individuals (Table 1), with the magnitude of this effect depending on both the severity of the MHW and the thermal affinity of the species (Figure 3).

In *L. digitata* , control and moderate‐MHW samples maintained high *F*_v_/*F*_m_ values until ~24 C–26 C, after which both showed clear declines (Figure 3). In contrast, individuals exposed to the extreme‐MHW began the experiment with already depressed *F*_v_/*F*_m_ values and showed marked declines at substantially lower temperatures (~20°C–22°C). Biomass loss and mortality also occurred earlier in the extreme‐MHW treatment, with samples becoming non‐viable at lower temperatures compared to the moderate‐MHW and control group. *S. latissima* displayed a similar but slightly more pronounced pattern (Figure 3). Control individuals remained physiologically healthy until ~26°C–28°C, whereas both moderate and extreme‐MHW treatments exhibited reduced initial *F*_v_/*F*_m_ values and steeper declines during the trial. Biomass loss began earlier in the moderate‐MHW treatment and was most severe in the extreme‐MHW treatment. Mortality thresholds occurred at lower temperatures in both MHW treatments, particularly the extreme exposure.

For the warm‐affinity species *L. ochroleuca* , the impacts of MHW exposure were substantial (Figure 3). Extreme‐MHW samples started the assay with heavily depressed *F*_v_/*F*_m_ values and exhibited rapid declines, reaching mortality by ~22°C–24°C. Moderate‐MHW samples showed intermediate responses, with declines beginning earlier than in controls but later than in the extreme treatment. Control individuals retained high *F*_v_/*F*_m_ until ~26°C–28°C and reached mortality only at ~30°C.

##### 3.2.2.1 CT_max_ Estimates

The logistic regression model showed that MHW exposure significantly reduced CT_max_ in all three species (Table 3), though the magnitude of reduction varied with thermal affinity (Table 2). In *L. digitata* , CT_max_ was significantly lower following the extreme‐MHW treatment (24.4°C ± 0.3°C) compared with both the control (27.1°C ± 0.6°C) and moderate‐MHW treatments (27.8°C ± 0.4°C). *S. latissima* displayed a similar response, with CT_max_ declining from the control (28.2°C ± 0.1°C) and moderate‐MHW (28.2°C ± 0.2°C) treatments to the extreme‐MHW exposure (24.0°C ± 0.1°C). In the warm‐affinity species *L. ochroleuca* , CT_max_ declined more sharply, decreasing significantly from the control (28.3°C ± 0.1°C) to the moderate‐MHW (27.8°C ± 0.2°C) to the extreme‐MHW treatment (20.2°C ± 0.4°C).

## 4 Discussion

Our results show that estimates of critical thermal maxima (CT_max_) vary substantially both between and within species, depending on thermal history, biological strategy and experimental context. This finding reinforces the growing view that CT_max_ is a plastic physiological property rather than a fixed species‐level trait (Lutterschmidt and Hutchison 1997; Bates and Morley 2020; Ørsted et al. 2022). Rather than undermining CT_max_ as a metric, the variability observed here provides mechanistic insight into how marine foundation species integrate past thermal exposure and physiological buffering capacity, and highlights that its application requires careful, context‐dependent interpretation when assessing resilience and vulnerability under ocean warming.

Excised meristematic discs are widely used in experimental seaweed ecophysiology, largely due to logistical constraints and the difficulty of working with large kelp plants (Hereward et al. 2020; Niedzwiedz et al. 2022; Leathers et al. 2023). In the present study, this approach proved appropriate for the cool‐water species *L. digitata* and *S. latissima*, where discs closely reproduced whole‐plant stress responses and yielded comparable CT_max_ estimates. In contrast, *L. ochroleuca* exhibited clear divergence between excised discs and whole plants, with discs significantly underestimating whole‐plant CT_max_. This difference is likely explained by species‐specific biological strategies rather than thermal affinity alone. *L. ochroleuca* is characterised by rapid seasonal growth and comparatively lower long‐term storage of carbon reserves, relying more strongly on whole‐plant physiological integration to maintain tissue performance under stress (Schiener et al. 2015; Pessarrodona et al. 2019). When meristematic tissue is excised, access to integrated internal resources and coordinated stress responses is lost, revealing a stronger dependence on whole‐organism buffering than in the cooler‐affinity species. As such, this finding does not undermine the use of excised discs but instead highlights the need to validate reduced tissue approaches on a species‐by‐species basis, as their ability to represent whole‐plant thermal tolerance depends on their underlying biology.

Thermal history across seasonal timescales had a pronounced influence on CT_max_ in the two cool‐water species. Both *L. digitata* and *S. latissima* exhibited significantly lower CT_max_ values in autumn compared to spring and summer, alongside earlier declines in photo‐physiological performance, greater biomass loss and earlier mortality. These patterns strongly suggest cumulative energetic stress accrued over summer reduces resilience later in the year, consistent with previous findings of intra‐annual variability in stress tolerance in kelp species (Hereward et al. 2020; Niedzwiedz et al. 2022; Straub et al. 2022). This seasonal effect is particularly relevant given that populations of *L. digitata* and *S. latissima* occur near the warm range edge of their biogeographic distribution in the WEC, where thermal safety margins are narrow and summer temperatures increasingly approach sub‐lethal thresholds (Smale et al. 2013). In contrast, the warm‐affinity, *L. ochroleuca* , occupies a leading‐edge position and showed only modest seasonal variation in CT_max_. Ambient seasonal temperatures in the study region likely remain closer to this species' thermal optimum, limiting cumulative stress accumulation and promoting ecophysiological performance throughout the year (Franco et al. 2018). Together, these patterns suggest that cool‐water kelps in this region are already exhibiting early signs of physiological stress accumulation, which may translate into heightened vulnerability with the intensification of stressful summer temperatures (Simon et al. 2023). In conjunction with seasonal cycles in temperature and other abiotic factors, kelp species exhibit seasonal phenology in terms of timings of reproduction, recruitment, growth and senescence (Kain 1989). As such, the reduced thermal tolerance in the latter part of the year, which follows the fast‐growth phase through spring into summer (Pessarrodona et al. 2019), may also relate to intra‐annual variability in biological processes and physiology.

Exposure to simulated MHWs significantly reduced CT_max_ in all three species, consistent with growing evidence that MHWs erode physiological tipping points in kelps and other seaweeds (Straub et al. 2022; Leathers et al. 2023; King et al. 2024). The magnitude of this response, however, differed markedly among species. Cool‐water species showed moderate reductions following extreme MHW exposure, reflecting reduced initial conditions and narrower thermal safety margins. In *L. ochroleuca* , CT_max_ surprisingly declined dramatically following extreme MHW exposure, decreasing by more than 7°C relative to controls. Interpreted alongside Experiment 1, this pronounced shift suggests that sustained thermal stress can overwhelm whole‐plant buffering mechanisms once internal resource pools are depleted, particularly when tissue is isolated.

The context‐dependent CT_max_ plasticity observed here mirrors responses reported across a wide range of marine foundation organisms (Osland et al. 2013; Kirwan and Megonigal 2013; Marin‐Guirao et al. 2017; Hughes et al. 2017; Schoepf et al. 2015), indicating that dynamic upper thermal limits are a common feature of these species. In reef‐building corals, for example, thermal thresholds shift with both seasonal climatology and exposure to MHWs, with tolerance often lowest following warm seasons or repeated heat stress that depletes internal energy reserves and compromises physiological buffering (Schoepf et al. 2015; Hughes et al. 2017). Similarly, seagrasses show reduced resilience later in the year following warm summers, and extreme heat events can further erode tolerance by exhausting carbohydrate stores and suppressing metabolic recovery, with effects that persist long after temperatures return to ambient conditions (Marin‐Guirao et al. 2017; Serrano et al. 2021). In vegetated coastal systems such as mangroves and saltmarsh plants, both seasonal warming and extreme thermal events can reduce subsequent resilience by altering energetic balance and stressing repair pathways (Kirwan and Megonigal 2013; Osland et al. 2013). Together, these studies demonstrate that both seasonal climatology and MHWs can drive lasting reductions in physiological resilience by altering energetic state and buffering capacity, reinforcing the interpretation of CT_max_ as a dynamic, history‐dependent trait rather than a fixed tolerance limit.

Overall, our findings highlight that CT_max_ is best interpreted as a flexible, history‐dependent trait, shaped by both organismal biology and environmental context. Incorporating this plasticity into vulnerability assessments will improve predictions of kelp forest resilience under future ocean warming.

## Author Contributions

**Tayla Leathers:** conceptualization (lead), data curation (equal), investigation (equal), methodology (equal), project administration (equal), visualization (lead), writing – original draft (lead), writing – review and editing (equal). **Dan A. Smale:** supervision (lead), writing – review and editing (supporting). **Nathan G. King:** formal analysis (equal), investigation (equal), methodology (equal), project administration (equal), supervision (equal), visualization (equal), writing – review and editing (equal).

## Conflicts of Interest

The authors declare no conflicts of interest.

## Supporting information


**Figure S1:** Illustrative diagram showing the experimental design of the study.
**Table S1:** Detailed information for all models used for *F*_v_/*F*_m_ for species and each experiment. The model with the lowest AIC was chosen.

## Data Availability

Data will be available from the Figshare repository https://doi.org/10.6084/m9.figshare.32123602.
