# Supplementary material for: Estimates of Critical Upper Maxima (CTmax ) of Kelp Species Are Strongly Influenced by Biogeography and Thermal History
Source: Ecol Evol. 2026 Sep 4;16(9):e74276. doi: 10.1002/ece3.74276 (PMC13545005; doi:10.1002/ece3.74276)
Supplement: Supplementary file 1 [file ECE3-16-e74276-s001.docx]

Figure S1: Illustrative diagram showing the experimental design of the study.

Table S1: Detailed information for all models used for Fv/Fm for species and each experiment. The model with the lowest AIC was chosen.

| Species | Experiment | Model | AIC |
| --- | --- | --- | --- |
| *L. digitata* | 1 | Fv/Fm ~ Temperature | 36.98 |
|  |  | Fv/Fm ~ Temperature + Treatment | 38.04 |
|  |  | Fv/Fm ~ Temperature * Treatment | 39.50 |
|  | 2 | Fv/Fm ~ Temperature | 159.06 |
|  |  | Fv/Fm ~ Temperature + Treatment | 106.67 |
|  |  | Fv/Fm ~ Temperature * Treatment | 95.66 |
|  | 3 | Fv/Fm ~ Temperature | 83.89 |
|  |  | Fv/Fm ~ Temperature + Treatment | 52.11 |
|  |  | Fv/Fm ~ Temperature * Treatment | 55.58 |
| *S. latissima* | 1 | Fv/Fm ~ Temperature | 34.61 |
|  |  | Fv/Fm ~ Temperature + Treatment | 36.30 |
|  |  | Fv/Fm ~ Temperature * Treatment | 38.16 |
|  | 2 | Fv/Fm ~ Temperature | 163.14 |
|  |  | Fv/Fm ~ Temperature + Treatment | 98.59 |
|  |  | Fv/Fm ~ Temperature * Treatment | 78.57 |
|  | 3 | Fv/Fm ~ Temperature | 75.81 |
|  |  | Fv/Fm ~ Temperature + Treatment | 51.20 |
|  |  | Fv/Fm ~ Temperature * Treatment | 54.98 |
| *L. ochroleuca* | 1 | Fv/Fm ~ Temperature | 3.65 |
|  |  | Fv/Fm ~ Temperature + Treatment | 1.79 |
|  |  | Fv/Fm ~ Temperature * Treatment | -1.13 |
|  | 2 | Fv/Fm ~ Temperature | -22.19 |
|  |  | Fv/Fm ~ Temperature + Treatment | -24.84 |
|  |  | Fv/Fm ~ Temperature * Treatment | -24.65 |
|  | 3 | Fv/Fm ~ Temperature | 139.62 |
|  |  | Fv/Fm ~ Temperature + Treatment | 50.01 |
|  |  | Fv/Fm ~ Temperature * Treatment | 43.30 |
